# A Liquid Crystal Ionomer‐Type Electrolyte toward Ordering‐Induced Regulation for Highly Reversible Zinc Ion Battery

**DOI:** 10.1002/advs.202206469

**Published:** 2023-01-16

**Authors:** Du Yuan, Xin Li, Hong Yao, Yuhang Li, Xiaobo Zhu, Jin Zhao, Haitao Zhang, Yizhou Zhang, Ernest Tang Jun Jie, Yi Cai, Madhavi Srinivasan

**Affiliations:** ^1^ College of Materials Science and Engineering Changsha University of Science and Technology Changsha Hunan 410004 P. R. China; ^2^ State Key Laboratory of Organic Electronics and Information Displays & Institute of Advanced Materials (IAM) Nanjing University of Posts & Telecommunications 9 Wenyuan Road Nanjing 210023 China; ^3^ Institute of Process Engineering Chinese Academy of Sciences Beijing 100190 China; ^4^ School of Chemistry and Materials Science Institute of Advanced Materials and Flexible Electronics (IAMFE) Nanjing University of Information Science and Technology Nanjing 210044 China; ^5^ School of Materials Science and Engineering Nanyang Technological University Block N4.150 Nanyang Avenue Singapore 639798 Singapore

**Keywords:** ionomer, liquid crystal electrolyte, ordering, zinc ion battery, Zn

## Abstract

Novel electrolyte is being pursued toward exploring Zn chemistry in zinc ion batteries. Here, a fluorine‐free liquid crystal (LC) ionomer‐type zinc electrolyte is presented, achieving simultaneous regulated water activity and long‐range ordering of conduction channels and SEI. Distinct from water network or local ordering in current advances, long‐range ordering of layered water channels is realized. Via manipulating water activity, conductivities range from ≈0.34 to 15 mS cm^−1^, and electrochemical window can be tuned from ≈2.3–4.3 V. The Zn|Zn symmetric cell with LC gel exhibits highly reversible Zn stripping/plating at 5 mA cm^−2^ and 5 mAh cm^−2^ for 800 h, with retained ordering of water channels. The capability of gel for inducing in situ formation of long‐range ordered layer SEI associated with alkylbenzene sulfonate anion is uncovered. V_2_O_5_/Zn cell with the gel shows much improved cycling stability comparing to conventional zinc electrolytes, where the preserved structure of V_2_O_5_ is associated with the efficiently stabilized Zn anode by the gel. Via long‐range ordering‐induced regulation on ion transport, electrochemical stability, and interfacial reaction, the development of LC electrolyte provides a pathway toward advancing aqueous rechargeable batteries.

## Graphical abstract

A liquid crystal zinc electrolyte is developed to regulate water activity and long‐range ordering of ion conduction channels and in situ SEI. The designed ordering‐induced regulation leads to promising cycling stability in V_2_O_5_/Zn ZIB with highly stable Zn anode.

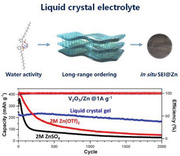

## 1 Introduction

As thrilling alternatives beyond lithium ion batteries, aqueous zinc ion batteries (ZIBs) are on their fast track. Utilizing metallic Zn anode endows ZIBs with the advantages of natural abundance, promising electrochemical performance, and environmental friendliness.^[^ ^1^ ^]^ However, the interfacial chemistry/electrochemistry of Zn anode is complexed by the critical factors of dendrite formation, competitive hydrogen evolution reaction (HER), and formation of solid‐state electrolyte (SEI), which largely hinders the pursuit of high‐performance ZIBs toward their applications.^[^ ^2^ ^]^ The above can alter the surface distribution of electric field, charge concentration and charge flux, disturbing the essence of achieving homogeneous Zn deposition.^[^ ^3^ ^]^ Besides advancing metallic anode design,^[^ ^4^ ^]^ novel zinc electrolytes and surface protection methods are intensely investigated toward highly reversible Zn anode.^[^ ^2^, ^5^ ^]^

The concept of “water‐in‐salt” electrolyte with extremely high concentration salt successfully extends the electrochemical window (EW) and prospers a wide range of aqueous battery systems,^[^ ^6^ ^]^ where the reconstructed zinc coordination in 1 m Zn(TFSI)_2_‐20m LiTFSI facilitates a reversible Zn stripping/plating with depressed HER.^[^ ^7^ ^]^ In parallel, ionic liquid based electrolyte (e.g., Zn(BF_4_)_2_ in [EMIM]BF_4_) and deep eutectic solvents (e.g., Zn(TFSI)_2_‐acetamide) are explored to suppress the water reactivity,^[^ ^8^ ^]^ through manipulating the coordination and hydrogen bonding (H‐bonding).^[^ ^9^ ^]^ However, the use of highly concentrated fluorinated salts raises the concern on environment and cost. Alleviating the fluorine concern, a molecular crowding network was designed with 2 m LiTFSI in poly(ethylene glycol) to stabilize the water molecules with a voltage window of 3.2 V.^[^ ^10^ ^]^ Alternatively, “water‐in‐ionomer” gel (e.g., 50% lithiated polyacrylic acid (LiPAA)‐50% H_2_O) was proposed.^[^ ^11^ ^]^ Compared with the highly concentrated fluorinated electrolytes, the relatively high water content in the LiPAA gel could restrict the EW and proceed the side reactions deteriorating cycling life. It is then critical to manipulate the water activity to achieve mobility and redox stability simultaneously. Importantly, ion/water conduction channel has the advantage of guiding Zn^2+^ transport and hence manipulating the reaction mechanism, which is usually achieved by proton/ion exchange membrane or surface coating.^[^ ^12^ ^]^ To construct ordered conduction channel, advancing material design is pursued.^[^ ^13^ ^]^ Therefore, it remains as a question for an electrolyte, whether the regulation of water activity can be extended from local‐ to long‐range ordering. In addition, high cation transference number benefiting ion transport and interface kinetics,^[^ ^14^ ^]^ implies the mobility of anion needs to be restricted, which sets further requirement on electrolyte design.

On the other hand, significance of SEI in aqueous rechargeable batteries is key‐noted.^[^ ^15^ ^]^ An ideal SEI for ZIB guides ion transport, hinders water‐associated side reaction, and facilitates dendrite‐free Zn deposition.^[^ ^2f^ ^]^ In parallel with surface protection layer on Zn as artificial SEI, electrolyte‐induced SEI possesses self‐generating characteristics favoring long‐term cycling,^[^ ^16^ ^]^ where the fundamental understanding on its formation mechanism is progressively studied. Anion‐derived nature of SEI has been revealed in the TFSI^−^‐based “water‐in‐salt” electrolyte and deep eutectic solvent, where the reductive decomposition of TFSI^−^ leads to the formation of ZnF_2_ and S/N‐rich organic compounds.^[^ ^3^, ^8^ ^]^ On the other hand, interfacial phase of zinc complex hydroxide has been identified in salt‐in‐water regime, e.g., ZnSO_4_·[Zn(OH)_2_]_3_·nH_2_O by ZnSO_4_, and Zn_x_(OTf)_y_(OH)_2x‐y_·nH_2_O by Zn(OTf)_2_.^[^ ^12^, ^17^ ^]^ The formation of complex hydroxide can be understood by the increase of local pH and prevailing existence of anion intercalated zinc hydroxide in neutral electrolyte, where the control on SEI growth has strong impact on the cycling lifetime of Zn anode. Cooperative mechanism of solvent decomposition and zinc complexation can also occur in ZnCl_2_‐H_2_O‐DMSO electrolyte forming Zn_12_(SO_4_)_3_Cl_3_(OH)_15_·5H_2_O.^[^ ^18^ ^]^ Presently, the ability for an electrolyte to induce an effective SEI on Zn draws special attention, where in situ SEI construction approach further complexes the interfacial chemistry.

Hence, the great challenge for developing zinc electrolyte lies in manipulating the water activity while depressing water‐associated reaction, rationalizing ion conduction pathway and regulating interfacial zinc chemistry. In this work, we demonstrate for the first time, a fluorine‐free liquid crystal (LC) type zinc electrolyte for ZIB, with regulated water state, and long‐range ordering of conduction channels and induced SEI. Through the control on the water content, a successive series of electrolytes were obtained from solid, wax, gel, to liquid state. Importantly, long‐range ordering of layered water channels is achieved in the lyotropic LC electrolyte, which is distinguishable from the water network or local ordering in current arts. The gel electrolyte brings the benefits of wide EW, high conductivity, and stable Zn stripping/plating, where the ordering of water channels can be well retained during cycling. An average CE of ≈100% was achieved. Meanwhile, a homogeneous layered SEI was revealed to be constructed in situ during cycling, which stabilizes the Zn anode at high current density, e.g., stable cycling at 5 mA cm^−2^ and 5 mAh cm^−2^ for 800 h. The merits further contribute to the greatly improved cycling performance in V_2_O_5_/Zn full cell compared with the commonly applied zinc electrolytes. We anticipate the significance of ordering‐induced regulation in electrolyte design.

## 2 Results and Discussion

### 2.1 LC Zinc Electrolyte

**Figure 1:**
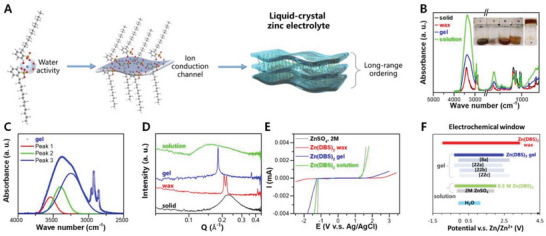
A) Scheme of LC zinc electrolyte, tuning water activity and constructing ion conduction channel. B) FTIR‐ATR spectra indicating the hydrogen bonding status in the Zn(DBS)_2_‐H_2_O electrolytes, where the vibrational peaks at 2958, 2922, 2872, and 2853 cm^−1^ can be assigned to the ‐CH_2_ group, the peaks at ≈1200 and 1047 cm^−1^ can be assigned to asymmetric and symmetric vibration of ‐SO_3_ respectively, and the one at 1602 cm^−1^ is assigned to the phenyl ring.^[^ ^20^, ^21^ ^]^ The inset shows the digital photo of a series of Zn(DBS)_2_‐H_2_O electrolytes for (i) solid, (ii) wax, (iii) gel and (iv) solution states, respectively. C) Deconvolution of the FTIR spectrum showing the states of water for the gel, where Peak 1, 2, 3 refer to the weakly‐bonded liquid‐like amorphous state, ice‐like liquid state, and ice‐like state of H_2_O, respectively. D) SAXS spectra presenting the evolving ordering of water channels in Zn(DBS)_2_‐H_2_O. E) LSV spectra of Zn(DBS)_2_‐H_2_O from solution to wax states, where the electrochemical window can be effectively widen toward both oxygen evolution and hydrogen evolution ends, with F) comparison on the reported EW values for the novel gel electrolytes in ZIBs.^[^ ^8^, ^22^ ^]^

Tailoring the hydrated Zn(H_2_O)_6_^2+^ coordination in aqueous electrolyte is typically approached by introducing polar zincophlilic anions/groups, solvents or additives, where surrounding H‐bonded network is altered. Little is dedicated to constructing long‐range water channel via re‐organizing H bond.^[^ ^19^ ^]^ Inspired by the structural ordering in the liquid crystal phase, we propose a novel zinc salt with long alkane chain and zincophilic sulfonate group (**Figure** **1**A,B, and Figure S1, Supporting Information). Intimately associated with structure change of lyotropic LC, the arrangement of water channels in zinc bis(2‐dodecylbenzenesulfonate) (Zn(DBS)_2_)‐H_2_O aqueous system is revealed by small‐angle x‐ray scattering (SAXS) in Figure 1D. As water content increases, the ordering of material is drastically altered. In its solid state (Zn(DBS)_2_·0.6H_2_O), a broad diffraction peak at ≈0.226 Å^−1^ represents the typical lamellar layering in Zn(DBS)_2_.^[^ ^20^ ^]^ A waxy state (Zn(DBS)_2_·5.4H_2_O) can be obtained through equilibrating at certain humidity (Figure S2). The doublets at 0.208 and 0.219 Å^−1^ can be assigned as water channel and stacked alkane chain layer, respectively, which could be related to the insufficient water to form alternative layering. Also, the apparent peak sharpening compared with its solid state suggests a water‐assisted crystallization of the alkane chain. Interestingly, Zn(DBS)_2_ gel (Zn(DBS)_2_·20H_2_O) shows a sharp single peak at ≈0.181 Å^−1^ (Q, an ordered spacing of 3.47 nm), indicating the formation of lamellar phase with alternative water and alkane chains layers. Higher ordered peaks at ≈0.362 (2Q) and 0.548 (3Q) Å^−1^ verifies the long‐range order of conduction channels in Zn(DBS)_2_ gel (Figure S3, Supporting Information). Further increase of water amount disturbs the ordering, showing an upshift of the much broaden diffraction feature of water channels, e.g., ≈0.150 Å^−1^for Zn(DBS)_2_·55.3H_2_O (equivalently, 0.5 m Zn(DBS)_2_ aqueous solution). Hence, by the stacking of long‐chain hydrophobic backbones with hydrophilic sulfonate groups, long‐range proton/ion conduction channels can be constructed in the Zn(DBS)_2_‐H_2_O system. We further employed polarized optical microscopy to observe the LC phase in Zn(DBS)_2_‐H_2_O (Figure S4, Supporting Information). Combined with the x‐ray diffraction patterns, the LC phase of Zn(DBS)_2_‐H_2_O changes from irregular lamellar (solid), to 1D lamellar (wax, gel), then to a mixture of lamellar and micellar (solution), and to irregular micellar structure (solution).

Concerning ion transportation, the conductivity of Zn(DBS)_2_‐H_2_O can vary from 0.34 to 15 mS cm^−1^ associated with the water content (Table S1, Supporting Information). 0.5 m Zn(DBS)_2_ aqueous solution shows comparable conductivity to those of the commonly applied ZnSO_4_ and Zn(OTf)_2_‐based aqueous electrolytes. And the conductivity of Zn(DBS)_2_ gel of ≈7 mS cm^−1^ facilitates its function as electrolyte. Also, Zn^2+^ transference number was evaluated to be as high as ≈0.75 for the gel in the symmetric Zn configuration (Figure S5, Supporting Information), compared with typically below 0.5 for conventional aqueous zinc electrolytes (Table S1, Supporting Information). The high transference number is possibly attributed to the immobilization of anion in the LC phase,^[^ ^14^ ^]^ which can effectively mitigate the concentration gradient and polarization, and decrease the ion transfer resistance at interface.^[^ ^23^ ^]^

Electrochemical windows were assessed to evaluate the stability of Zn(DBS)_2_‐H_2_O (Figure 1E). As increasing the salt concentration, the onset potential of oxygen evolution was positively shifted by ≈2.0 V; the onset of hydrogen evolution was negatively shifted by ≈0.7 V. Overall, EW can extend from 2.3 V for 0.5 m Zn(DBS)_2_ (solution) to 2.7 V for Zn(DBS)_2_·20H_2_O (gel), and further to 4.3 V for Zn(DBS)_2_·5.4 H_2_O (wax). Compared with pure water (theoretical EW of 1.23 V) and the conventional aqueous zinc electrolyte (e.g., EW of ≈2.0 V for 2 m ZnSO_4_), both oxygen evolution and hydrogen evolution can be successfully depressed through manipulating the water content in of Zn(DBS)_2_‐H_2_O, where the EW of Zn(DBS)_2_ gel is among the highest reported values for the water‐containing gel electrolytes (Figure 1F).^[^ ^8^, ^22^ ^]^

To better understand the state of water in the Zn(DBS)_2_‐H_2_O electrolyte system, the broad ‐OH stretching feature was deconvoluted to study the detailed H‐bonding structure (Figure 1D and Figure S6, Supporting Information). Three species of H_2_O molecules are differentiated, weakly‐bonded liquid‐like amorphous H_2_O (≈3540 cm^−1^), ice‐like liquid H_2_O with tetrahedral H‐bonded network (≈3400 cm^−1^), and ice‐like H_2_O with tetrahedral H‐bonded network (≈3230 cm^−1^).^[^ ^24^ ^]^ Concentrating the salt leads to the reducing relative population of liquid‐like amorphous H_2_O (from 15.6% in 0.5 m solution to only 2.4% in the solid state) and enhancing relative population of ice‐like liquid H_2_O (from 21.9% in 0.5 m solution to 36.5% in the solid state), with that of ice‐like H_2_O little changed (Figure S7, Supporting Information). In addition, both bonding states of ice‐like liquid H_2_O and ice‐like H_2_O exhibited overall high‐frequency shifts of ≈11 and ≈107 cm^−1^ respectively when being concentrated (Figure S8, Supporting Information). This is consistent with the shift of ‐OH bending from 1635 cm^−1^ in solution to1653 cm^−1^ in solid state. The blue shifts of both stretching and bonding of ‐OH can be attributed to the strengthened H_2_O‐SO_3_^−^ interaction, which is consistent with the blue shift of ‐SO_3_ symmetric vibration from the solution (≈1035 cm^−1^) toward solid state (≈1047 cm^−1^). Thus, more than contributing to the conduction channels, the presence of ‐SO_3_‐ group leads to the reconstruction of the H‐bonding network. The ordered state, e.g., ice‐like H_2_O, is associated with the ion transfer and separation in the Volmer step during HER,^[^ ^24^, ^25^ ^]^ where the corresponding blue‐shift vibration might suggest a higher overpotential for HER. Also, the enlarged percentage of ice‐like liquid component suggests more water in the ordered state. Along with the observed blue‐shift, it indicates more energy required to electrochemically decompose water in the presence of ‐SO_3_^−^ with the increasing salt concentration. Consequently, the needed higher overpotential for oxygen evolution or hydrogen evolution indicates the enhanced electrochemical stability as the observed widen EWs in Zn(DBS)_2_‐H_2_O.

### 2.2 Reversible Zn Stripping/Plating with in situ Induced SEI

**Figure 2:**
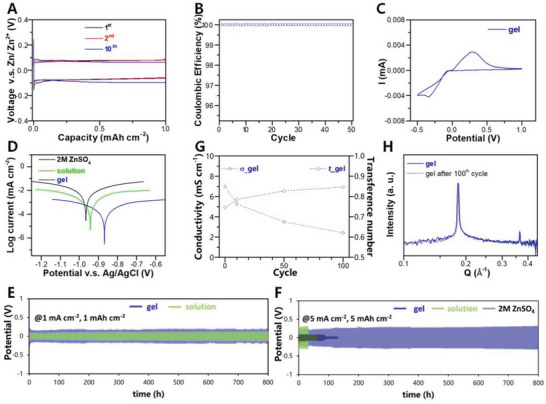
A) Polarization curves of Ti|Zn asymmetric cell with Zn(DBS)_2_ gel under a galvanostatic capacity of 1 mAh cm^−2^ and 0.5 mA cm^−2^, with B) the long term CEs. C) Cyclic voltammetry curve of Zn stripping/plating in Zn(DBS)_2_ gel at a scan rate of 1 mV s^−1^. D) Tafel plots comparing the corrosion behavior of Zn in Zn(DBS)_2_ solution, gel and 2 m ZnSO_4_. E) Galvanostatic Zn stripping/plating in the Zn|Zn symmetric cells for Zn(DBS)_2_ solution and gel at 1 mA cm^−2^ and 1 mAh cm^−2^. F) Stable Zn stripping/plating of the Zn|Zn cell with Zn(DBS)_2_ gel at 5 mA cm^−2^ and 5 mAh cm^−2^ for 800 h, compared the cells with 2 m ZnSO_4_ and Zn(DBS)_2_ solution. Real‐time investigation on Zn(DBS)_2_ gel during Zn stripping/plating for the first 100 cycles: G) Change of conductivities (*σ*) and transference numbers (*t*_Zn_^2+^) with cycling, H) ex situ SAXS comparison on the ordering of water channel before and after cycling.

To investigate the reversibility of Zn stripping/plating in Zn(DBS)_2_ gel, Coulombic efficiency was investigated in asymmetric Ti|Zn cell with a galvanostatic capacity of 1 mAh cm^−2^ at 0.5 mA cm^−2^ (**Figure** **2**A). The CE quickly approaches 100% within the first few cycles and retains well at ≈100% over 50 cycles (Figure 2B), which demonstrates the high reversibility of the gel in stripping/plating. Note that an overpotential of ≈157 mV is required for the 1^st^ cycle in the gel, while ≈102 mV is needed in the following cycles. It suggests a progressively improved stability at the electrolyte‐Zn interface. The redox feature associated with Zn stripping/plating can be pictured though Cyclic voltammetry (CV) (Figure 2C), where the CE analysis further supports the highly reversible stripping/plating behavior in the gel (Figure S9, Supporting Information). In addition, Zn(DBS)_2_ gel exhibits excellent anticorrosion capability (Figure 2D). The observed positive shift of the corrosion potential of Zn in Zn(DBS)_2_ gel (≈‐0.87 V v.s. Ag/AgCl) compared with that in ZnSO_4_ (≈‐0.96 V v.s. Ag/AgCl) suggests less tendency toward corrosion. Meanwhile, the significantly reduced corrosion current in the gel indicates a reduced corrosion rate. To further study the long‐term effects of the Zn(DBS)_2_‐based electrolytes on Zn, the lifespan of Zn stripping/plating was evaluated in a Zn|Zn symmetric configuration using commercial Zn foils. Despite of relatively larger polarizations (≈228 and 443 mV, respectively) (Figure S10, Supporting Information), the symmetric cells with both Zn(DBS)_2_ solution and gel present stable stripping/plating curves over 800 h at 1 mA cm^−2^ (Figure 2E). At an elevated current density of 5 mA cm^−2^, the cell with Zn(DBS)_2_ gel shows an impressive stability over 800 h, in contrast with the cell with ZnSO_4_ (≈84 h) or with 0.5 m Zn(DBS)_2_ (≈30 h) (Figure 2F). The above distinctly demonstrates the advantage of Zn(DBS)_2_ gel electrolyte toward stabilizing Zn anode.

It is worth noting that the change of gel properties are during Zn stripping/plating is rarely assessed, and hence, a real‐time study on Zn(DBS)_2_ gel was performed at 1 mA cm^−2^ and 1 mAh cm^−2^ before polarization stablized (≈100 cycles). A decrease of gel conductivity can be observed once stripping/plating starts (Figure 2G), where such trend slows down after 50 cycles and a value of ≈2.5 mS cm^−1^ can be retained after 100 cycles. Meanwhile, the transference number increases gradually from 0.75 to 0.85 (Figure 2H). By ex situ FTIR (Figure S11, Supporting Information), a water amount equivalent to ≈n = 16.6 was estimated. Ex situ SAXS shows that the gel still possesses the feature of 1D lamellar structure after 100 cycles, where the first order diffraction peak shifts to a higher Q of ≈0.186 Å^−1^, corresponding to a slightly reduced ordered spacing of 3.38 nm (compared with 3.47 nm for the pristine state). The evolving transport behavior can be related to the changing water content, shrunk layer spacing of water channel, and resritced anion motion.

**Figure 3:**
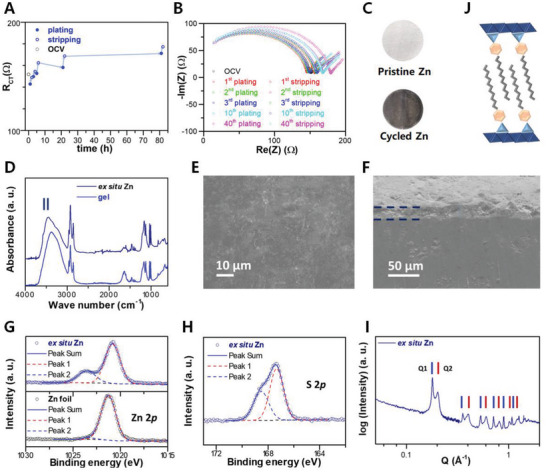
A) Time‐dependent study on the charge transfer resistance (R_CT_) of Zn anode with the gel electrolyte during the initial 40 stripping/plating cycles, where the arrows provide visual guide for the process. The values of R_CT_ were extracted from B) the corresponding decoupled EIS spectra for each step. C) Optical image of Zn foil before and after cycling. D) SEM image of the cycled Zn foil with E) cross section study showing the formation of surface layer. F) FTIR of the cycled Zn foil showing the vibrational features of ‐CH_2_, phenyl, ‐SO_3_^−^ groups and water, with emerging ‐OH group. XPS spectra of the cycled Zn for G) Zn2*p* and H) S2*p*, respectively. I) GISAXS spectrum of the cycled Zn showing the long‐range ordering of surface groups. J) Scheme showing the induced SEI formed by the proposed complex zinc hydroxide.

Importantly, the graduate change of polarization in the symmetric cell (Figure 2E and 2F) suggests the evolving electrolyte‐Zn interface that warrants elaboration. Electrochemical impedance spectroscopy (EIS) was applied to monitor the electrolyte‐Zn interface during cycling. Perceiving that both working and counter electrodes contribute to the EIS of a symmetric cell, the recorded spectrum was decoupled using a customized 3‐electrode cell, such that the change of impedance during sequential plating and stripping steps for an individual Zn electrode can be uncovered. **Figure** **3**A,B shows that the charge transfer resistance increases from ≈143 Ω at 1^st^ plating to ≈152 Ω at 3^rd^ plating, and gradually to ≈171 Ω at 40^th^ plating, indicating a higher energy barrier for deposition with accumulating steps. A notable higher charge transfer resistance is observed for the adjacent stripping in each corresponding cycle. The above could reflect the growth of SEI during stripping, which brings higher resistance for interfacial kinetics, influencing the deposition process. Moreover, the increasing trends toward plateaus for both plating and stripping processes may further support the stabilization of the electrolyte‐Zn interface.

Corresponding characterizations were employed to explore the material nature of the induced SEI. Ex situ optical microscopy shows a homogenous dark layer on the Zn foil after stripping/plating at 1 mA cm^−2^ and 1 mAh cm^−2^ for 400 cycles (Figure 3C). SEM study indicates the formation of a uniform surface layer (Figure 3D), with a thickness of ≈24 µm (Figure 3E). Vibrational spectral features of ‐CH_2_ vibration (2958, 2924, 2872, and 2854 cm^−1^), phenyl group vibration (1600 cm^−1^), and ‐SO_3_^−^ group (1200 and 1035 cm^−1^ for asymmetric and symmetric modes, respectively) indicate that the surface layer may consist of alkane chain and sulfonate group. The retention of water can be also viewed by its bending (≈1650 cm^−1^) and stretching bands (≈3750 to 2600 cm^−1^) (Figure 3F). Besides resembling the vibrational features of Zn(DBS)_2_, the spectrum presents emerging peaks at ≈3580 and 3445 cm^−1^, which can be attributed to the presence of ‐OH groups as in complex hydroxides.^[^ ^26^ ^]^ Elemental analysis was conducted on the surface (Figure 3G and 3H). With reference to metallic Zn (≈1021.3 eV) with oxidation (≈1023.0 eV), ex situ Zn shows an emerging peak of ≈1023.7 eV, which can be attributed to Zn‐OH.^[^ ^27^ ^]^ Meanwhile, S2*p* is deconvoluted into peaks at ≈167.3 and 168.5 eV, which reflects the bonding of ‐SO and its surrounding H_2_O molecules.

To shed light on the molecular arrangement of the surface layer, grazing incidence small angle x‐ray scattering (GISAXS) was applied. Two series of diffraction peaks were detected (Figure 3I), starting from ≈0.18 Å^−1^ (Q1) and 0.20 Å^−1^ (Q2) with their corresponding higher order diffraction peaks (Table S2, Supporting Information). The spacings for Q1 (≈35.0 Å) and relatively broad Q2 (≈31.0 Å) can be attributed to the hydroxide layer and alignment of anion with alkane chain respectively, where the difference between the spacings is consistent with the layer thickness of the zinc‐based hydroxide (≈4.6 Å).^[^ ^28^ ^]^ The larger spacing for Q2 than the size of DBS anion (≈24.8 Å) suggests the interpenetrating model for nearly vertical chain alignment in DBS‐incorporated hydroxide, favoring charge balance in the material.^[^ ^28^, ^29^ ^]^ Meanwhile, the arrangement can facilitate the ion transport through the conduction channels formed by the aligned sulfonate groups. Thus, the above clearly indicates the long‐range ordering in the surface layer, as supported by the XRD spectrum (Figure S12, Supporting Information). Based on the aforementioned analysis, we propose this surface layer to be the DBS‐intercalated complex zinc hydroxide,^[^ ^26^, ^29^ ^]^ i.e., Zn(DBS)_x_(OH)_y_·nH_2_O. The complex zinc hydroxide can be formed during the stripping step when Zn^2+^ ions are released, which then lead to the increase on the resistance of charge transfer at the interface (Figure 3A). Meanwhile, the enhancement on the intensity ratio for Zn (002)/(101) indicates the favorable formation of (002) facilitating stable stripping/plating, which can be understood from the anion induced effects through sulfonate group as we previously reported.^[^ ^4c^ ^]^

Based on the above, the LC electrolyte is able to induce the in situ formation of SEI (Figure 3J), where the layer structure is constructed by the folding of long alkane chains with zincophilic sulfonate group. Inheriting the characteristics of the LC electrolyte, the induced SEI may bring the merits of inhibiting HER by the uniform hydrophobic chains, facilitating the transportation of zinc ions and further guiding the stripping/plating process through the conduction channels endowed with the sulfonate groups, and thus stabilizing Zn anode.

### 2.3 Long‐Term Stable Cycling of ZIB with LC Gel

**Figure 4:**
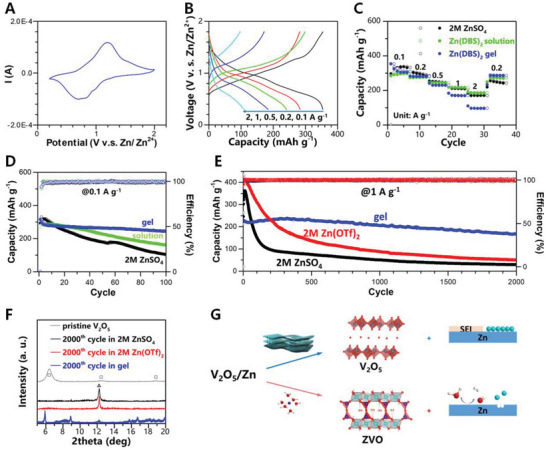
Electrochemical properties of V_2_O_5_/Zn(DBS)_2_/Zn cells. A) CV profile of V_2_O_5_/Zn(DBS)_2_ gel/Zn cell at a scan rate of 1 mV s^−1^, with B) GCD curves of the cell at a series of current densities from 0.1 to 2 A g^−1^. C) Rate capabilities for the cells with different electrolytes. Comparison on the cycling stability of the cells with different electrolytes at D) 0.1 A g^−1^ and E) 1 A g^−1^, respectively. F) ex situ XRD patterns of V_2_O_5_ cathodes at charged state in different electrolytes after 2000^th^ cycle at 1 A g^−1^, where the crystallographic phases are indicated for V_2_O_5_ (□) and ZVO (△). G) Scheme showing the gel electrolyte stabilizing the cycling of V_2_O_5_/Zn, where side reaction on Zn as in the conventional aqueous electrolyte is largely hindered, leading to the well‐retained V_2_O_5_ structure.

We further investigate the viability of Zn(DBS)_2_ gel for ZIB. V_2_O_5_/Zn was selected as the model full cell, where the deteriorated capacity during cycling is associated with Zn corrosion‐related V_2_O_5_ dissolution in aqueous media.^[^ ^30^ ^]^ It is worth anticipating the influence of the gel electrolyte on the cell. A typical CV profile of the full cell in **Figure** **4**A shows the distinct pairs of redox peaks, indicating the Zn^2+^ intercalation/deintercalation processes as reported in mixed‐valence V_2_O_5_.^[^ ^31^ ^]^ Consistently, the galvanostatic charge‐discharge (GCD) curves of the cell with Zn(DBS)_2_ gel at 0.1 A g^−1^ exhibit profiles with two sloping discharge/charge regions at ≈0.7 and 1.1 V respectively, delivering a capacity of ≈360 mAh g^−1^ (Figure 4B, and also the cell with Zn(DBS)_2_ solution in Figure S13, Supporting Information). With increasing charging/discharging rates, the cell with Zn(DBS)_2_ solution presents higher capacities than that with ZnSO_4_ (Figure 4C). When current density is switched back to low value (0.2 A g^−1^), a ≈100% retention of the capacity is achieved for the cell with the gel electrolyte, compared with only ≈80% for the cell with ZnSO_4_. The cycling stability of the V_2_O_5_/Zn cell with Zn(DBS)_2_ was evaluated. At 0.1 A g^−1^, ≈77% of the capacity can be well retained for the gel electrolyte with ≈100% CE after 100 cycles (Figure 4D). Similarly, capacity retention of ≈76% can be achieved by using Zn(DBS)_2_ solution. In sharp contrast, only ≈35% of the capacity can be retained with ZnSO_4_. At a high current density of 1 A g^−1^ after 2000 cycles, a great retention of ≈75% can be achieved with Zn(DBS)_2_ gel (Figure 4E), which is superior to ≈11% retention with ZnSO_4_ and ≈13% retention with Zn(OTf)_2_.

Ex situ XRD shows that after long‐term cycling in ZnO_4_ or Zn(OTf)_2_, the cathode at charged state can barely retain its crystal structure compared with the layer structure in the pristine mixed‐valence V_2_O_5_ (6.53° for (001) diffraction, corresponding to an interlayer spacing of 13.5 Å) (Figure 4F). Instead, a narrow diffraction peak at ≈12.25° emerged, corresponding to zinc pyrovanadate (001) (ZVO, Zn_3_V_2_O_7_(OH)_2_·*n*H_2_O) (see higher order diffraction features in Figure S14, Supporting Information). However, for the electrode cycled in gel electrolyte, the diffraction of the layer structure is clearly detected and shifted to ≈5.92° (corresponding to an enlarged interlayer spacing of 14.9 Å), together with the weak ZVO phase. Such phase change is further reflected in ex situ FTIR‐ATR spectra (Figure S15, Supporting Information). The electrode retains well the vibrational characteristics of VOBV stretching and apical VOA in‐plane stretching with the gel, in contrary to diminishing features with either ZnSO_4_ or Zn(OTf)_2_. Recently, the dissolution of V_2_O_5_ in aqueous environment is perceived as the dominating factor responsible for the capacity decay during cycling.^[^ ^30^ ^]^ Through dissolution, the produced VO_2_(OH)_2_^−^ and H^+^ leads to the formation of electrochemically inactive ZVO between VO_2_(OH)_2_^−^ and Zn^2+^, where the consumption of released H^+^ via Zn corrosion can render the reaction. Consequently, a well‐retarded Zn corrosion can effectively shift the balance of V_2_O_5_ dissolution and improve the cycling stability of ZIB. Note that both H^+^ and Zn^2+^ can co‐function in V_2_O_5_/Zn cell with Zn(DBS)_2_ gel, leading to the change of interlayer spacing and diffusion coefficients of ≈2.3 × 10^−11^ to 4.3 × 10^−10^ cm^2^ s^−1^ during discharge (Figure S16, Supporting Information). This can be supported by comparing V_2_O_5_/Zn cell in organic solvent with depressed proton production, where a discharged capacity of ≈115 mAh g^−1^ at 0.1 A g^−1^ was obtained by using Zn(DBS)_2_‐DMF (molar ratio of 1:20) (Figure S16, Supporting Information). Moreover, a surface structure is uncovered on the cathode by ex situ SAXS showing the ordering of complex hydroxide (≈0.18 Å) and alkylchain (≈0.21 Å) (Figure S17, Supporting Information). Together with ATR analysis (Figure S18, Supporting Information), it can be attributed to the DBS^−^ incorporated structure as above discussed. This might bring further protection to the cathode due to the hydrophobicity of alkane chain.

Hence, the excellent cycling stability of the cell with Zn(DBS)_2_ gel is attributed to the followings (Figure 4G). Through manipulating the Zn^2+^ coordination with the activity of water, Zn(DBS)_2_ gel possesses high electrochemical stability and can effectively depress hydrogen evolution, leading to reversible Zn stripping/plating. The induced SEI consisting of well‐arranged alkane chain as protective layer and sulfonate group regulating ion conduction, can stabilize Zn anode, contrary to the poorly protected Zn covered with the loosely packed SEI, such as in ZnSO_4_.^[^ ^12a^ ^]^ The much‐improved anti‐corrosion characteristics can hinder the consumption of released H^+^ and drive the chemical equilibrium toward retaining the structure of V_2_O_5_, endowing stable cycling with the gel electrolyte.

## 3 Conclusion

In summary, we have demonstrated a novel liquid‐crystal type Zn(DBS)_2_ electrolyte for ZIB. The condensed form of electrolyte can be controlled through adjusting the water content. Via manipulating the intermolecular interaction, activity of water can be effectively tuned, influencing its physiochemical properties such as widen electrochemical windows (2.3–4.3 V) and promising conductivities (0.34 to 15 mS cm^−1^). The molecular design of long‐alkane chain sulfonate group facilitates constructing conduction channels, where the LC gel electrolyte of Zn(DBS)_2_·20H_2_O shows a long‐range ordering of layered water channels (Q ≈0.181 Å^−1^). Also, a high transference number of ≈0.75 is achieved in the gel state, attributed to the restricted anion mobility. The gel electrolyte exhibits highly reversible Zn stripping/plating at 5 mA cm^−2^ and 5 mAh cm^−2^ for 800 h, superior to its solution counterpart or conventional ZnSO_4_. The stable Zn stripping/plating is associated with an in situ formed SEI, which is revealed during the successive stripping process by decoupling EIS. The structure of SEI is possibly attributed to Zn(DBS)_x_(OH)_y_·*n*H_2_O, where the long‐range alignment of DBS^−^ can lead to the formation of conduction channels and hydrophobic alkane chain can efficiently inhibit the side reactions. When applied in V_2_O_5_/Zn full cells, a much improved cycling stability of ≈75% retention can be achieved with the gel at 1 A g^−1^ after 2000 cycles, compared with below 13% retention for either ZnSO_4_ or Zn(OTf)_2_. The promising cycling stability is attributed to the well‐retained layer structure of V_2_O_5_ after cycling, and further associated with the gel electrolyte stabilized Zn anode. Hence, the developed LC electrolyte enables regulating ion transport, electrochemical stability, and interfacial reactions. From the perspective of ordering‐induced regulation on interface chemistry/electrochemistry, we anticipate the proposed design can promote the development of emerging electrolyte toward advance design in rechargeable batteries.

## 4 Experimental Section

### Synthesis of Zn(DBS)_2_ Based Electrolytes

Zinc dodecylbenzenesulfonate (Zn(DBS)_2_) was synthesized based on a modified protocol from previous report.^[^ ^20^ ^]^ Briefly, stochiometric ratio of ZnO was dissolved in dodecylbenzenesulfonic acid solution of isopropanol and ethanol at 70 °C for 2 h to obtain a clear solution. The crystallization process was carried on through solvent evaporation. To facilitate the control of water content, vacuum drying at 100 °C for 12 h was applied to obtain the solid phase of Zn(DBS)_2_.

A series of Zn(DBS)_2_‐H_2_O electrolytes were prepared. The solid was monitored under a humidity of ≈55% for a few days to achieve a steady state, and the water amount was determined by TGA. Based on the observation, a corresponding amount of water was added to prepare the waxy state. For Zn(DBS)_2_ gel and solution, corresponding amounts of water was added with the designed molar ratio.

### Materials Characterizations

Thermogravimetric analysis (TGA) was applied to determine the water content in the solid and waxy states. Fourier‐transform infrared spectroscopy measurement was conducted by Frontier MIR spectrometer (Shimadzu Trace‐100) with the attenuated total reflectance (ATR) compartment. Small‐wide angle X‐ray scattering was performed by SWAXS Nano‐inXider (Xenocs), where the liquid and gel samples were sealed in borosilicate glass, and the wax and solid samples were by customized Kapton‐sealed holder to prevent water loss under vacuum. X‐ray diffraction (XRD) patterns were obtained by using a Bruker D8 Advance powder diffractometer with Cu‐K*α* radiation (*λ* = 1.5406 Å). SEM study was performed by FESEM 7600F (JEOL) equipped with an Electron back Scattering detector and EDS detector (Oxford). XPS spectra were collected by Kratos AXIS Supra XPS with dual anode (Al/Ag K*α*) X‐ray monochromatic source.

### Electrochemical Characterizations

Linear sweep voltammetry (LSV) was applied to determine the EWs of electrolyte by Biologic potentiostat/galvanostat workstation SP‐200 in a 3‐electrode configuration, with Pt as working electrode, leakless type Ag/AgCl as reference electrode, and the commercial Zn foil as the counter electrode. Chronocoulometry (CV) curve of the electrolyte was collected in a 3‐electrode configuration or a coin cell configuration. Asymmetric Ti|Zn cells for long‐term cycling under a constant capacity of 1 mAh cm^−2^ and a current density of 0.5 mA cm^−2^.^[^ ^32^ ^]^ The ionic conductivity (*σ*) was calculated based on the following Equation, $\sigma =\frac{l}{RS}$, where R is the bulk resistance obtained by EIS, *l* is the distance between Pt foils, and *S* is the electrolyte‐foil contact area. For the solution phase, a conductivity meter (METTLER TOLEDO FE38) was used to calibrate using standard electroltyes.

### Zn Stripping/Plating

Zn|Zn symmetric cells were assembled in CR2016 configuration. The electrolytes included 2 m ZnSO_4_, Zn(DBS)_2_ gel and 0.5 m solution. The stripping/plating cycling was performed by a Neware battery testing system. Galvanostatic mode was applied under current densities of 1 mA cm^−2^ and 5 mA cm^−2^, respectively. To study the interface, electrochemical impedance spectroscopy (EIS) was measured on Biologic SP‐200 in a 3‐electrode configuration, with frequency ranging from 1k to 50 M Hz and an AC amplitude of 10 mV. For ex situ study, the cells were disassembled to collect the zinc papers after cycling. The Zn foils were washed by D. I. water repeatedly, soaked in D. I. water, and dried for the further characterizations.

### V_2_O_5_/Zn Full Cell

V_2_O_5_ nanosheets were synthesized by the previously reported process in large scale and the electrode preparation was conducted based on the previous report.^[^ ^31a^ ^]^ Briefly, the slurry was formulated by the synthesized V_2_O_5_ and Super P (TIMCAL) in a weight ratio of 8:2. After casting and vacuum drying, the binder‐free electrode on stainless steel mesh was with a mass loading of active material ≈1.5‐2 mg cm^−2^. The coin cell was then assembled using the V_2_O_5_ electrode (12 mm), electrolytes, glassy fiber separator (19 mm), and commercial Zn foil in CR2016 configuration. The galvanostatic charge/discharge curves were measured in the potential range of 0.3‐1.8 V by Neware battery tester.

## Conflict of Interest

The authors declare no conflict of interest.

## Supporting information

Supporting Information

## Data Availability

The data that support the findings of this study are available in the supplementary material of this article.

## References

[1] a) L. E. Blanc , D. Kundu , L. F. Nazar , Joule 2020, 4, 771;

[2] a) Q. Yang , Q. Li , Z. Liu , D. Wang , Y. Guo , X. Li , Y. Tang , H. Li , B. Dong , C. Zhi , Adv. Mater. 2020, 32, 2001854; 10.1002/adma.202001854 33103828

[3] Q. Yang , G. Liang , Y. Guo , Z. Liu , B. Yan , D. Wang , Z. Huang , X. Li , J. Fan , C. Zhi , Adv. Mater. 2019, 31, 1903778. 10.1002/adma.201903778 31517400

[4] a) J. Zheng , Q. Zhao , T. Tang , J. Yin , C. D. Quilty , G. D. Renderos , X. Liu , Y. Deng , L. Wang , D. C. Bock , C. Jaye , D. Zhang , E. S. Takeuchi , K. J. Takeuchi , A. C. Marschilok , L. A. Archer , Science 2019, 366, 645; 31672899 10.1126/science.aax6873

[5] a) C. Liu , X. Xie , B. Lu , J. Zhou , S. Liang , ACS Energy Lett. 2021, 6, 1015;

[6] a) L. Suo , O. Borodin , T. Gao , M. Olguin , J. Ho , X. Fan , C. Luo , C. Wang , K. Xu , Science 2015, 350, 938; 26586759 10.1126/science.aab1595

[7] F. Wang , O. Borodin , T. Gao , X. Fan , W. Sun , F. Han , A. Faraone , J. A. Dura , K. Xu , C. Wang , Nat. Mater. 2018, 17, 543. 29662160 10.1038/s41563-018-0063-z

[8] a) L. Ma , S. Chen , N. Li , Z. Liu , Z. Tang , J. A. Zapien , S. Chen , J. Fan , C. Zhi , Adv. Mater. 2020, 32, 1908121; 10.1002/adma.201908121 32091149

[9] a) H. Bi , X. Wang , H. Liu , Y. He , W. Wang , W. Deng , X. Ma , Y. Wang , W. Rao , Y. Chai , H. Ma , R. Li , J. Chen , Y. Wang , M. Xue , Adv. Mater. 2020, 32, 2000074; 10.1002/adma.202000074 32130746

[10] J. Xie , Z. Liang , Y.‐C. Lu , Nat. Mater. 2020, 19, 1006. 32313263 10.1038/s41563-020-0667-y

[11] X. He , B. Yan , X. Zhang , Z. Liu , D. Bresser , J. Wang , R. Wang , X. Cao , Y. Su , H. Jia , C. P. Grey , H. Frielinghaus , D. G. Truhlar , M. Winter , J. Li , E. Paillard , Nat. Commun. 2018, 9, 5320. 30552314 10.1038/s41467-018-07331-6 PMC6294254

[12] a) D. Yuan , W. Manalastas Jr , L. Zhang , J. J. Chan , S. Meng , Y. Chen , M. Srinivasan , ChemSusChem 2019, 12, 4889; 31475452 10.1002/cssc.201901409

[13] a) C. Yang , Q. Wu , W. Xie , X. Zhang , A. Brozena , J. Zheng , M. N. Garaga , B. H. Ko , Y. Mao , S. He , Y. Gao , P. Wang , M. Tyagi , F. Jiao , R. Briber , P. Albertus , C. Wang , S. Greenbaum , Y.‐Y. Hu , A. Isogai , M. Winter , K. Xu , Y. Qi , L. Hu , Nature 2021, 598, 590; 34671167 10.1038/s41586-021-03885-6

[14] K. D. Fong , J. Self , B. D. McCloskey , K. A. Persson , Macromolecules 2021, 54, 2575.

[15] Y. Yamada , J. Wang , S. Ko , E. Watanabe , A. Yamada , Nat. Energy 2019, 4, 269.

[16] J. Hao , L. Yuan , Y. Zhu , M. Jaroniec , S.‐Z. Qiao , Adv. Mater. 2022, n/a, 2206963. 10.1002/adma.202206963 36073668

[17] a) J. Hao , X. Li , S. Zhang , F. Yang , X. Zeng , S. Zhang , G. Bo , C. Wang , Z. Guo , Adv. Funct. Mater. 2020, 30, 2001263;

[18] L. Cao , D. Li , E. Hu , J. Xu , T. Deng , L. Ma , Y. Wang , X.‐Q. Yang , C. Wang , J. Am. Chem. Soc. 2020, 142, 21404. 33290658 10.1021/jacs.0c09794

[19] J. Lim , K. Park , H. Lee , J. Kim , K. Kwak , M. Cho , J. Am. Chem. Soc. 2018, 140, 15661. 30358996 10.1021/jacs.8b07696

[20] J. Ruokolainen , J. Tanner , G. ten Brinke , O. Ikkala , M. Torkkeli , R. Serimaa , Macromolecules 1995, 28, 7779.

[21] O. Ikkala , J. Ruokolainen , G. ten Brinke , M. Torkkeli , R. Serimaa , Macromolecules 1995, 28, 7088.

[22] a) J. Zhao , K. K. Sonigara , J. Li , J. Zhang , B. Chen , J. Zhang , S. S. Soni , X. Zhou , G. Cui , L. Chen , Angew. Chem., Int. Ed. 2017, 56, 7871; 10.1002/anie.201704373 28503917

[23] a) X. Zeng , J. Mao , J. Hao , J. Liu , S. Liu , Z. Wang , Y. Wang , S. Zhang , T. Zheng , J. Liu , P. Rao , Z. Guo , Adv. Mater. 2021, 33, 2007416; 10.1002/adma.202007416 33576130

[24] a) Z. Wang , A. Pakoulev , Y. Pang , D. D. Dlott , J Phys Chem A 2004, 108, 9054;

[25] N. Dubouis , A. Serva , R. Berthin , G. Jeanmairet , B. Porcheron , E. Salager , M. Salanne , A. Grimaud , Nat. Catal. 2020, 3, 656.

[26] J. Demel , J. Hynek , P. Kovář , Y. Dai , C. Taviot‐Guého , O. Demel , M. Pospíšil , K. Lang , J. Phys. Chem. C 2014, 118, 27131.

[27] a) J. M. Wu , Y.‐R. Chen , J. Phys. Chem. C 2011, 115, 2235;

[28] E. L. Crepaldi , P. C. Pavan , J. Tronto , J. B. Valim , J. Colloid Interface Sci. 2002, 248, 429. 16290548 10.1006/jcis.2002.8214

[29] H. Zhang , X. Wen , Y. Wang , J. Solid State Chem. 2007, 180, 1636.

[30] Y. Kim , Y. Park , M. Kim , J. Lee , K. J. Kim , J. W. Choi , Nat. Commun. 2022, 13, 2371. 35501314 10.1038/s41467-022-29987-x PMC9061739

[31] a) J. Zhao , H. Ren , Q. Liang , D. Yuan , S. Xi , C. Wu , W. Manalastas , J. Ma , W. Fang , Y. Zheng , C.‐F. Du , M. Srinivasan , Q. Yan , Nano Energy 2019, 62, 94;

[32] L. Ma , M. A. Schroeder , O. Borodin , T. P. Pollard , M. S. Ding , C. Wang , K. Xu , Nat. Energy 2020, 5, 743.

