# Supplementary material for: A Liquid Crystal Ionomer‐Type Electrolyte toward Ordering‐Induced Regulation for Highly Reversible Zinc Ion Battery
Source: Adv Sci (Weinh). 2023 Jan 16;10(8):2206469. doi: 10.1002/advs.202206469 (PMC10015864; doi:10.1002/advs.202206469)
Supplement: Supplementary file 1 — Supporting Information [file ADVS-10-2206469-s001.pdf]

ADVANCED SCIENCE
Open Access

Supporting Information

for *Adv. Sci.*, DOI 10.1002/advs.202206469

A Liquid Crystal Ionomer-Type Electrolyte toward Ordering-Induced Regulation for Highly Reversible Zinc Ion Battery

Du Yuan, Xin Li, Hong Yao, Yuhang Li, Xiaobo Zhu, Jin Zhao*, Haitao Zhang*, Yizhou Zhang, Ernest Tang Jun Jie, Yi Cai and Madhavi Srinivasan**

Supporting Information

A liquid crystal ionomer-type electrolyte towards ordering-induced regulation for highly reversible zinc ion battery

Du Yuan[1,*], *Xin Li*[1], *Hong Yao*[1], *Yuhang Li*[1], *Xiaobo Zhu*[1], *Jin Zhao*[2,*], *Haitao Zhang*[3,*], *Yizhou Zhang*[4], *Ernest Tang Jun Jie* [5], *Yi Cai*[5], *Madhavi Srinivasan*[5,*]

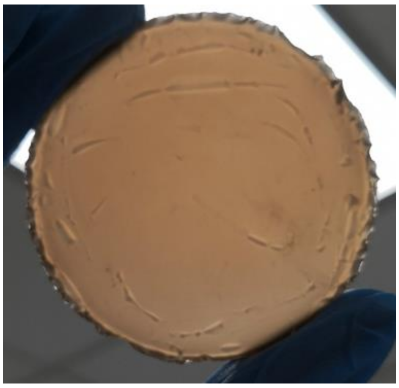

Figure S1. Optical image of as-synthesized $Zn(DBS)_2$ solid.

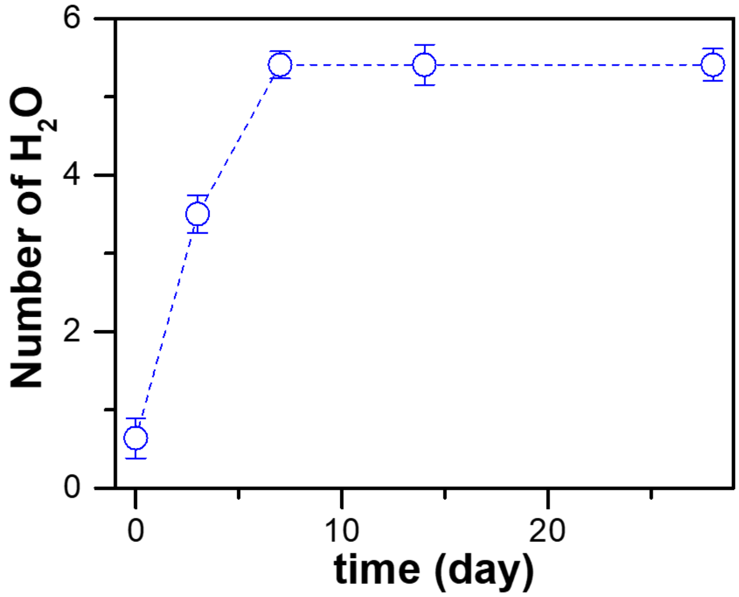

Figure S2. Change of water amount in $Zn(DBS)_2$ solid with time under a humidity of ~55%. The water content was determined by TGA and presented as n ($Zn(DBS)_2$:H_2O=1:n). The value of n quickly increased within 7 days from ~0.6 to ~5.4, and stabilized afterward within 28 days.

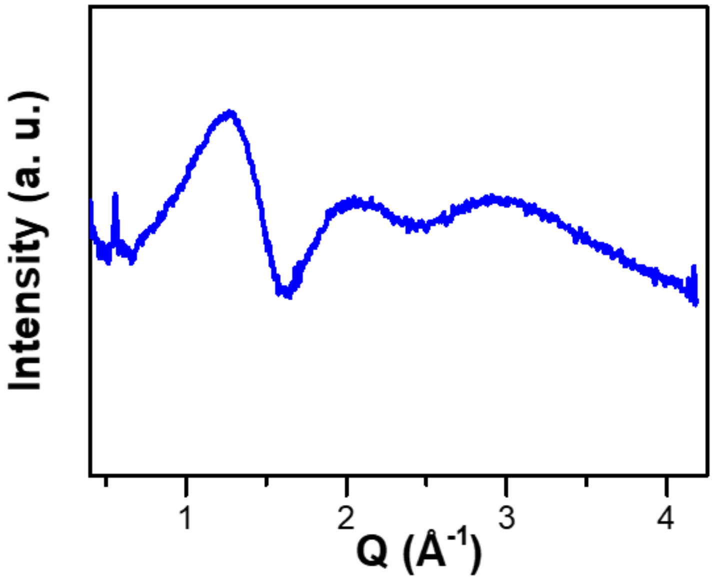

Figure S3. WAXS spectrum for $Zn(DBS)_2$ gel, where the sharp peak at ~0.548 $Å^{-1}$ corresponds to a diffraction order of 3Q regarding the layer structure.

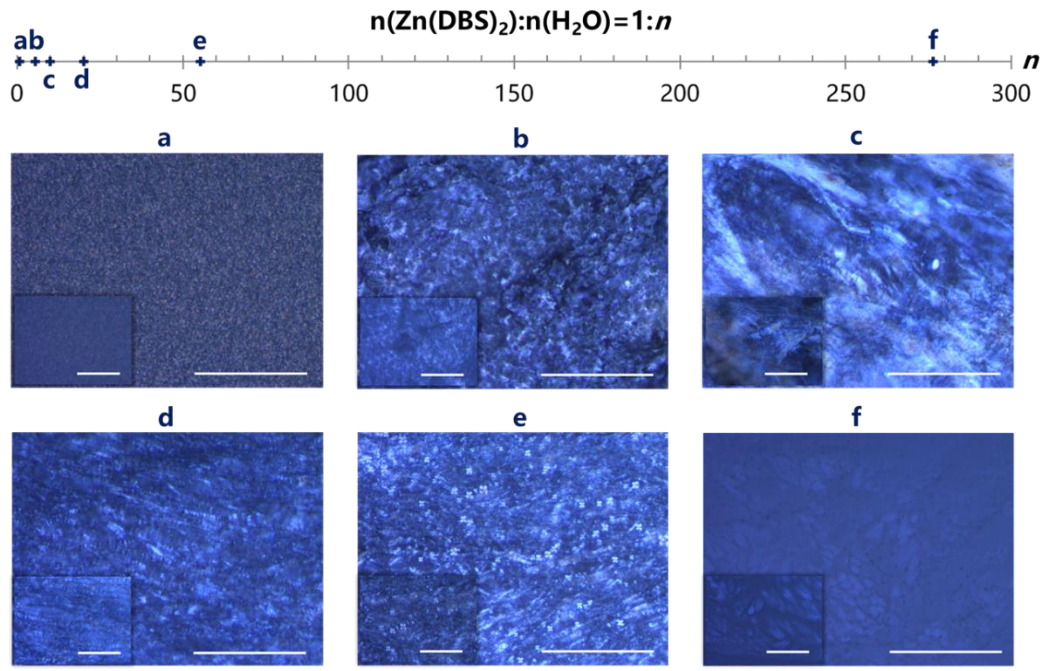

Figure S4. Phase study of $Zn(DBS)_2$-H_2O with changing water content (n($Zn(DBS)_2$:nH2O=1:*n*) by POM. The scale bars for the image and the corresponding inset are 100 and 50 µm, respectively.

To investigate the change of phase associated with the water content, polarized optical microscopy (POM) study was conducted. When *n* is 0.6 (a), irregular lamellar structure with weak ordering can be seen. At *n* is 5.4 (b), lamellar structure becomes clear. When *n* is between 10-20 (c, d), distinct lamellar structure is present. At *n*~55 (e), i.e., 0.5 M, dispersion of micellar phase in lamellar structure is found. When *n* further increases to ~277 (f), i.e., 0.1 M, irregular micellar units can be seen.
Moreover, the spacing ratio corresponding to diffraction peaks is used to determine the specific structural type of LC. According to the x-ray scattering data (Figure 1, Manuscript, and Figure S3, Supporting Information), the wax and gel phase can be attributed to 1D lamellar phase. For the solution, higher order diffraction is vague. The above is consistent with the reported structure for $Zn(DBS)_2$ (*Macromol. Rapid Commun. 2003, 24, 556*, *Macromolecules 1995, 28, 7779*).
From above, the POM observation is consistent with the SAXS spectra for $Zn(DBS)_2$-H_2O.

Table S1. Conductivities of $Zn(DBS)_2$ electrolytes with the transference numbers, compared with commonly applied zinc electrolytes.

electrolyte	σ(S cm^{-1})	Transference number
2 M $ZnSO_4$	0.054	~0.10
2 M $Zn(CF_3SO_3)_2$	0.069	~0.31
0.5 M $Zn(DBS)_2$	0.015	~0.55
$Zn(DBS)_2 \cdot 20H_2O$	0.007	~0.75
$Zn(DBS)_2 \cdot 5.4H_2O$	0.00034	-

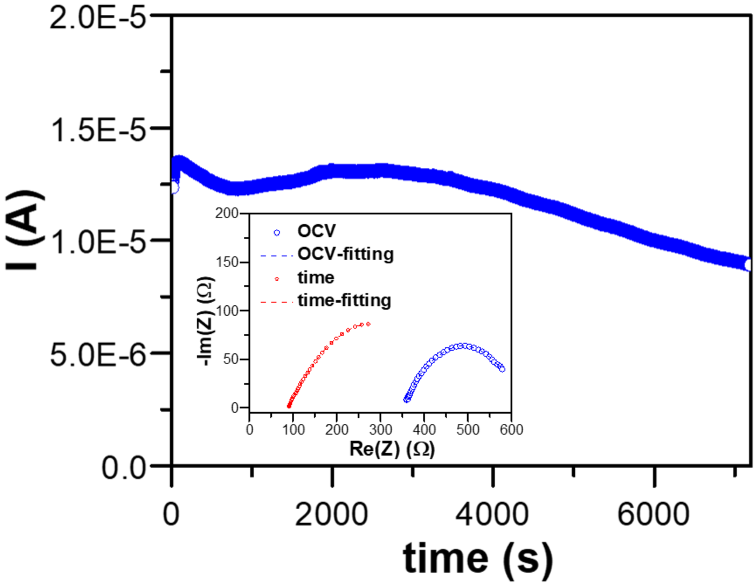

Figure S5. Current-time curves of the Zn symmetric cell with $Zn(DBS)_2$ gel after a constant potential of 10 mV for 7200 s. The inset shows the EIS spectra before and after polarization. From chronoamperometry and impedance data, the Zn^{2+} transference number ($t_{Zn^{2+}}$) was calculated by the following equation[1]:

$$t_{Zn^{2+}} = \frac{I_s(\Delta V - I_0 R_0)}{I_0(\Delta V - I_s R_s)},$$

Where ΔV is the applied constant polarization (10 mV), I_0 and R_0 are the initial current and resistance, and I_s and R_s are the steady-state current and resistance, respectively.

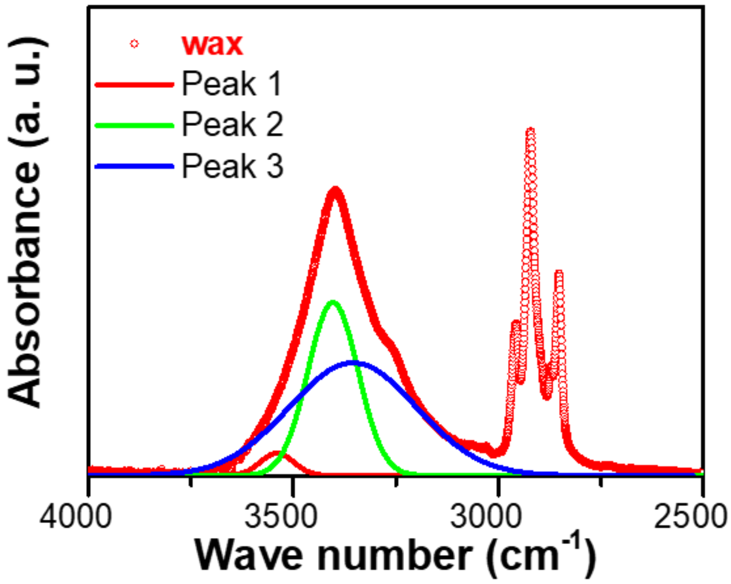

Figure S6. Peak deconvolution of FTIR spectra for $Zn(DBS)_2$ wax. Peak 1, 2, 3 refer to the weakly-bonded liquid-like amorphous state, ice-like liquid state, and ice-like state of H_2O, respectively.

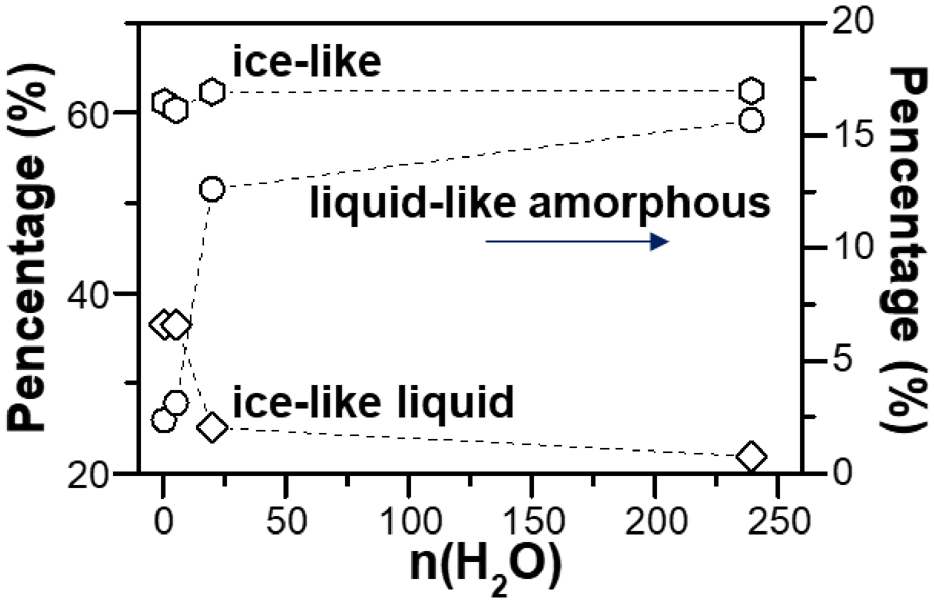

Figure S7. Relative percentages of the different water states across the $Zn(DBS)_2$ samples.

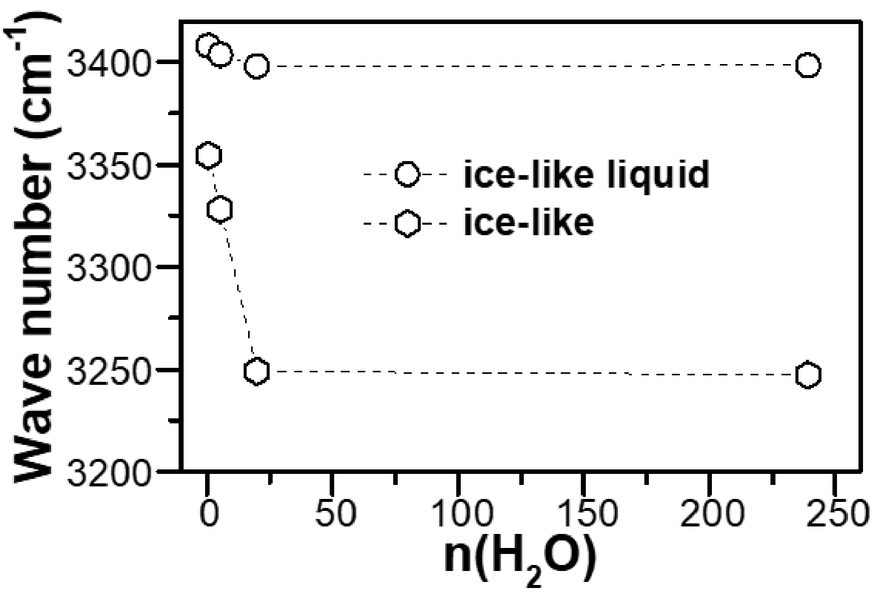

Figure S8. The change of vibrational frequencies associated with the ordered states of H_2O across the $Zn(DBS)_2$ samples.

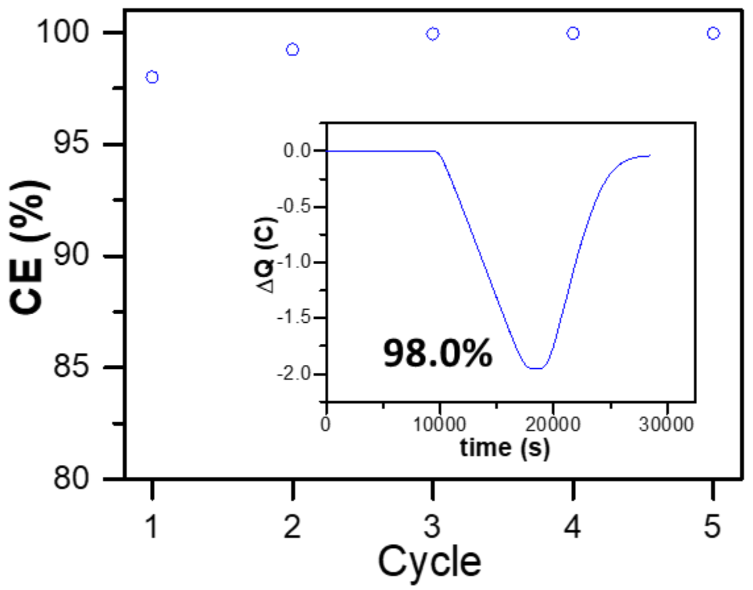

Figure S9. CEs of $Zn(DBS)_2$ gel estimated from its corresponding CV curves for the first 5 cycles, where the CE can reach a high CE of 98.0% at the 1st cycle and soon approach ~100%. The inset presents the 1st cycle chronocoulometry curve for CE calculation.

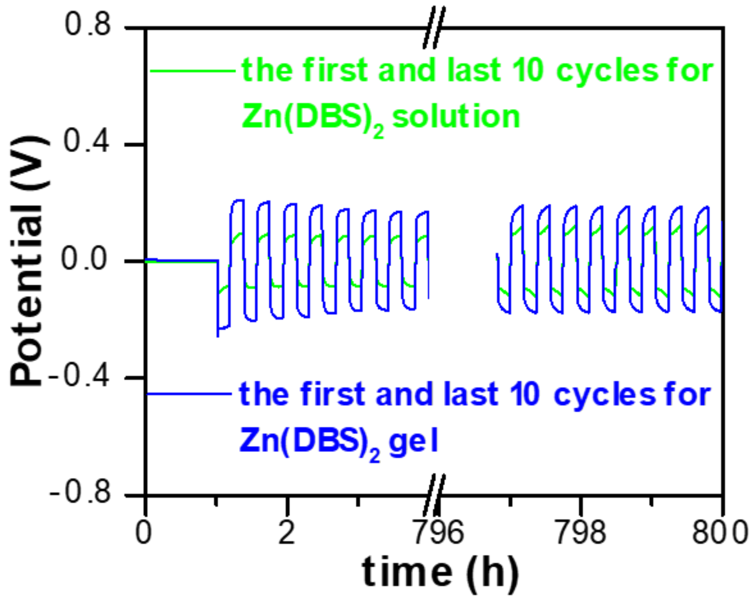

Figure S10. The voltage profiles for $Zn(DBS)_2$ solution and gel in Zn|Zn configuration under stripping/plating at 1 mA cm^{-2} and 1 mAh cm^{-2}.

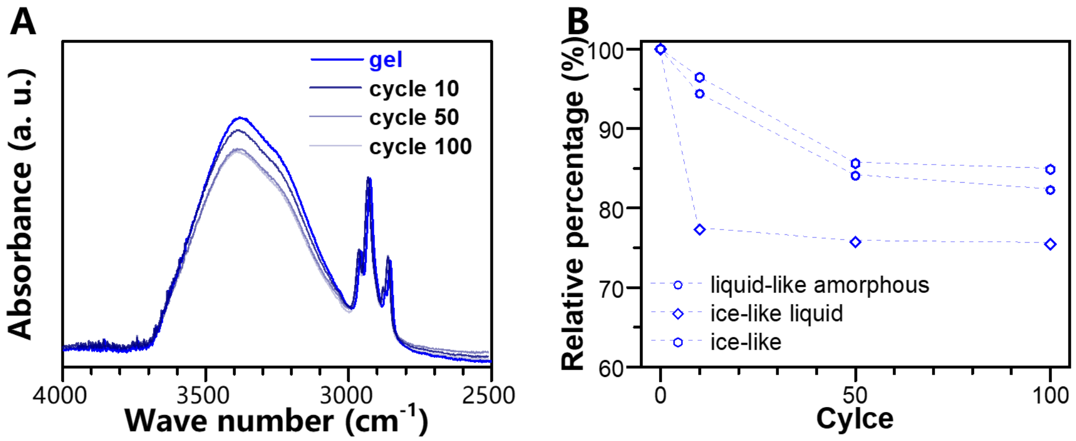

Figure S11. Real-time investigation on the gel electrolyte during Zn stripping/plating for the first 100 cycles: (A) FTIR-ATR spectra, and (B) the deconvoluted water states with cycling.

Perceiving the relation between water content/state and ion transport (Figure 1), sampling on water states during cycling was conducted by *ex situ* FTIR-ATR. By using the integrated intensity of vibrational bands from 4000 to 2500 cm^{-1}, the estimated water amount reduces to ~91.4% after 5 cycles, and till ~82.9% after 100 cycles (equivalently, n~16.6). Deconvolution of the spectra shows that the content of ice-like liquid (~3400 cm^{-1}) drops quickly after 5 cycles till ~77.3%, and further stabilizes at ~75.5% after 100 cycles. While the other two components of liquid-like amorphous (~3540 cm^{-1}) and ice-like (~3250 cm^{-1}) exhibit gradual change throughout cycling.

Table S2. Diffraction peaks obtained from GISAXS spectrum of cycled Zn ssigned to Q1 and Q2, respectively.

Q (Å^{-1})	2theta (deg)	Label
0.180	2.521	Q1
0.203	2.850	Q2
0.356	4.997	2Q1
0.399	5.599	2Q2
0.534	7.507	3Q1
0.606	8.512	3Q2
0.713	10.020	4Q1
0.813	11.430	4Q2
0.898	12.640	5Q1
1.012	14.254	5Q2
1.077	15.164	6Q1
1.212	17.088	6Q2

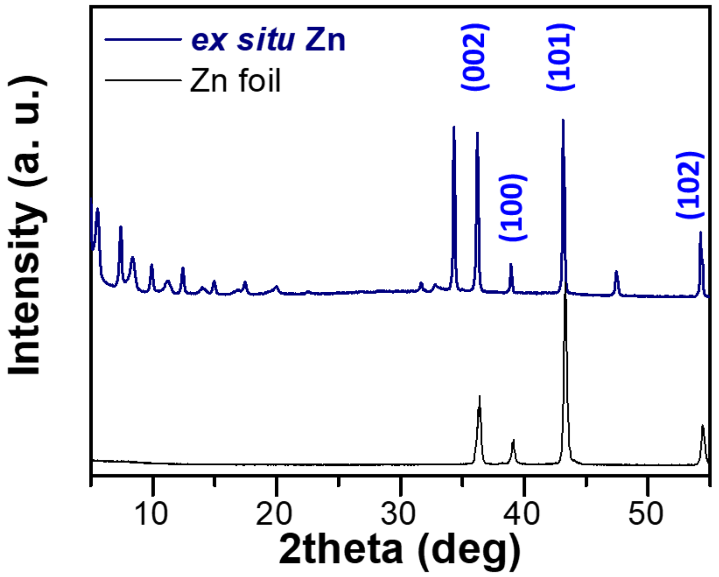

Figure S12. *ex situ* XRD pattern of Zn showing higher order diffractions for Q1 and Q2, confirming the long-range ordering in the formed surface layer during stripping/plating. Compared with the pristine Zn foil, a higher intensity ratio of (002)/(101) is observed, which can favor the reversible Zn stripping/plating.

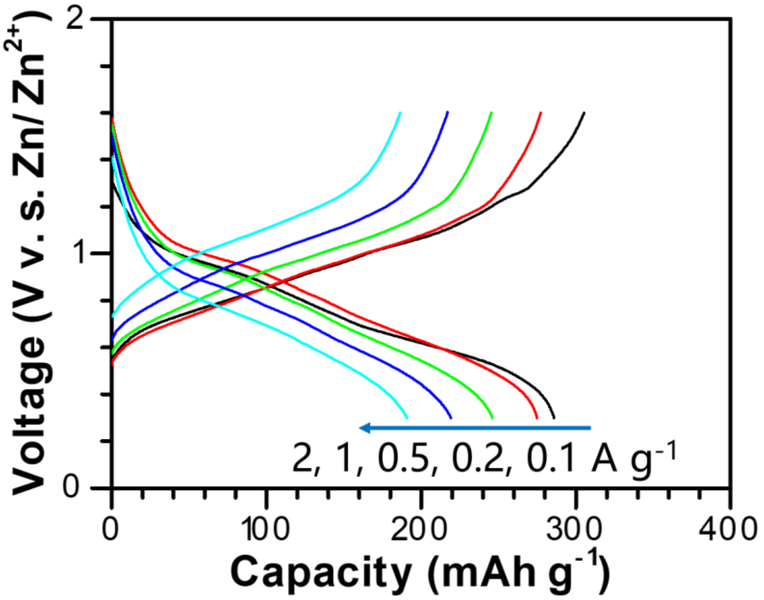

Figure S13. GCD curves of V_2O_5/Zn(DBS)$_2$ solution/Zn cell at a series of current densities from 0.1 to 2 A g^{-1}.

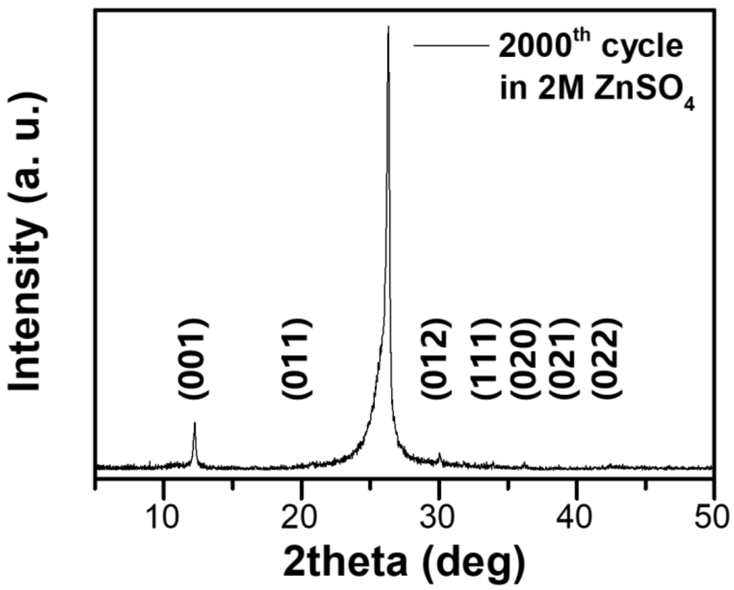

Figure S14. *ex situ* XRD spectrum for V_2O_5 cathode cycled in 2M $ZnSO_4$ at 1A g^{-1} for 2000 cycles, where the diffraction peaks are assigned to ZVO.

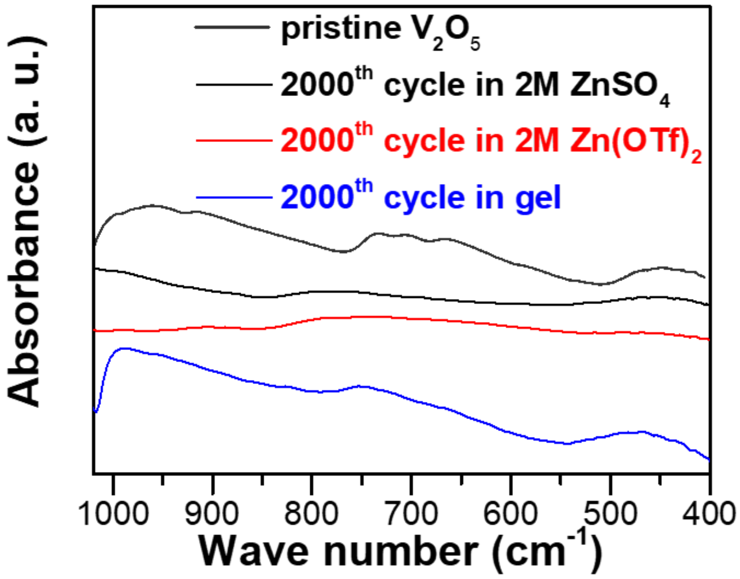

Figure S15. *ex situ* ATR spectra of the cathodes after cycling. In the pristine V_2O_5, vibrational peaks of ~453, 705, 980 cm^{-1} are assigned to VOBV stretching, VOC stretching, and apical VOA in-plane stretching modes, respectively[2]. The presence of 705 and 912 cm^{-1} reflects the reduced state of V^{5+}, which is consistent with the synthesized mixed-valence V_2O_5 [3]. After cycling either in 2M $ZnSO_4$ or 2M $Zn(OTf)_2$, the signals for V_2O_5 diminish, supporting the structure change of V_2O_5 as in *ex situ* XRD study (Figure 4F). An emerging weak peak at ~890 cm^{-1} is detected, which can be assigned to the formation of ZVO phase[4]. On the other hand, the cathode after cycling in $Zn(DBS)_2$ gel clearly presents the vibrational feature at ~453 and 980 cm^{-1}, consistent with the well retained structure of V_2O_5 as in **Figure 4F**. Meanwhile, the depressed signals at 705 and 912 cm^{-1} can be ascribed to the charged state of the samples, where V^{5+} valence dominates.

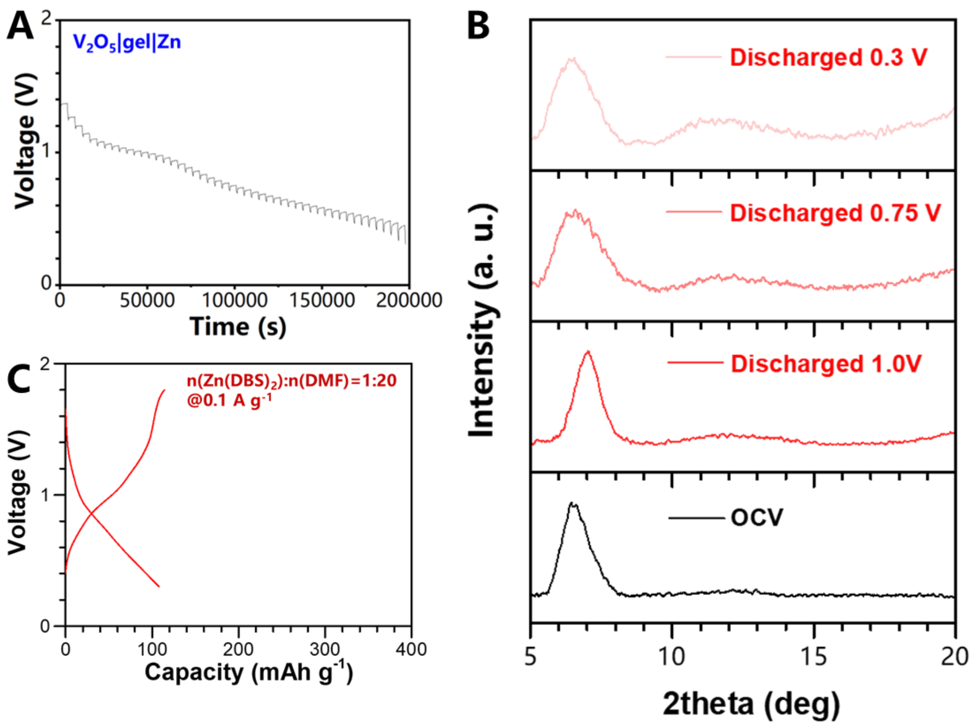

Figure S16. (A) Discharge GITT at 0.05 A g^{-1} for the V_2O_5/gel/Zn cell. (B) *ex situ* XRD for V_2O_5/gel/Zn at different discharged states at 0.1 A g^{-1}. (C) Charge/discharge curve of V_2O_5/Zn using $Zn(DBS)_2$-DMF electrolyte at 0.1 A g^{-1}.

For Galvanostatic intermittent titration technique (GITT) measurement, the V_2O_5/Zn cell with $Zn(DBS)_2$ gel was discharged at 0.05 A g^{-1} for 10 min and then relaxed for 1 h to make the voltage reach the equilibrium. During the discharge process, the calculated D is ~2.3×10^{-11} to 4.3×10^{-10} cm^2 s^{-1}. The D range is consistent with current report, where both H^+ and Zn^{2+} play their roles (*ACS Energy Lett. 2018, 3, 1366*, *Nano Energy 2019, 62, 94*).
According to *ex situ* XRD spectra, the diffraction peak at ~6.53° of V_2O_5 (001) shifts to a higher angle of ~7.02° when discharged to 1 V, indicating a reduced interlayer spacing (~12.6 Å). When being further discharged, the diffraction angle shifts to lower degree of ~6.57° at 0.75 V and till ~6.46 ° (corresponding to an interlayer spacing of ~13.7 Å) at 0.3 V. The reduced interlayer spacing of the V_2O_5 (001) diffraction peak might be attributed to the insertion of cation, while the increased interlayer spacing can be associated with the water-assisted cation insertion (*Nat. Commun. 2018, 9, 1656*, Zn^{2+}, *ACS Energy Lett. 2018, 3, 1366, Adv. Mater. 2017, 1703725*, *Nat. Energy 2016, 1, 16119*, *J. Am. Chem. Soc. 2017, 139, 9775*). Also, an emerging peak of ~8.8° can be detected when discharged at 0.3 V. It might be attributed to that during discharging, Zn^{2+} is driven towards cathode, facilitating the growth of solid-electrolyte interface of zinc complex hydroxide. After cycling, such interface can be viewed more noticeably (**Figure S17** and **S18**).
To further verify the charge storage behavior, an organic solvent based $Zn(DBS)_2$ electrolyte was prepared to depress the contribution from proton. A molar ratio of $n(Zn(DBS)_2)$:n(DMF)=1:20 was used, which has the equivalent molar ratio of salt over solvent as in $Zn(DBS)_2$ gel. The V_2O_5/Zn cell with $Zn(DBS)_2$-DMF presents a discharging capacity of ~115 mAh g^{-1} at 0.1A g^{-1}. This supports the above inference that both H^+ and Zn^{2+} co-function during charging/discharging. Such lower discharge capacity compared with ~135 mAh g^{-1} from 0.75 to 0.3 V with the gel electrolyte, might be attributed to the relatively sluggish kinetics of zinc salt in organic solvent.

From the above, it shows that the charge storage in V_2O_5/gel/Zn is associated with co-functioning of H^+ and Zn^{2+}, which is consistent with current literature.

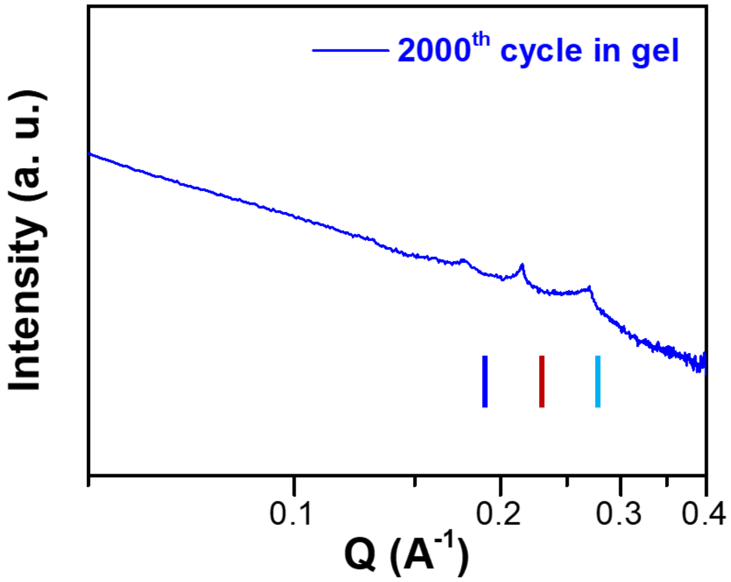

Figure S17. *ex situ* SAXS spectrum of V_2O_5 cycled at gel electrolyte after 2000th cycle, where Q at ~0.18 and 0.21 Å^{-1} can be assigned to the ordering of water channel and alkylchain, respectively. Also, the broad Q at ~0.24-0.28 Å^{-1} might be attributed to a higher layering of V_2O_5 structure (corresponding to a layer spacing of ~25 Å).

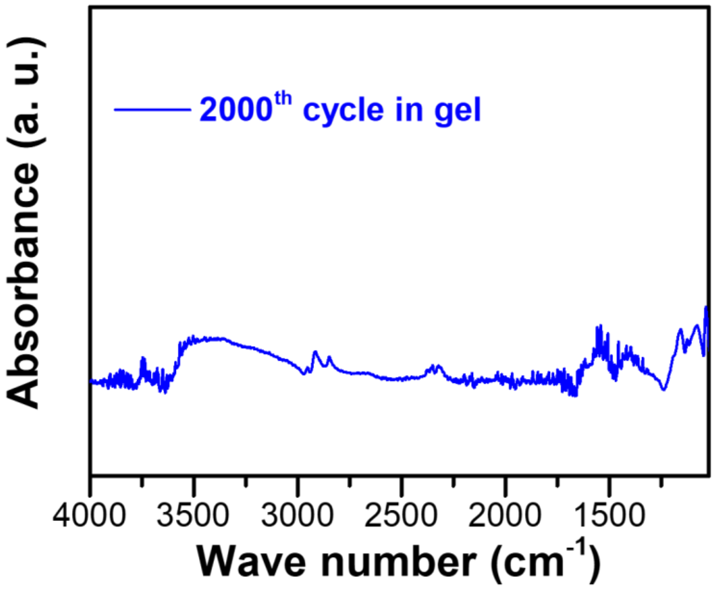

Figure S18. *ex situ* ATR spectrum for the cathode cycled in the gel. The vibration frequencies from 3000-2800, ~1600, and ~1035 cm^{-1} are attributed the -CH_2, phenyl ring, and symmetric vibration of -SO_3^- respectively.

References

[1] a)J. Evans, C. A. Vincent, P. G. Bruce, *Polymer* **1987**, 28, 2324; b)F. Wu, N. Chen, R. Chen, Q. Zhu, J. Qian, L. Li, *Chemistry of Materials* **2016**, 28, 848.
[2] a)L. Abello, E. Husson, Y. Repelin, G. Lucazeau, *Spectrochimica Acta Part A: Molecular Spectroscopy* **1983**, 39, 641; b)R. Baddour-Hadjean, J. P. Pereira-Ramos, C. Navone, M. Smirnov, *Chemistry of Materials* **2008**, 20, 1916.
[3] J. Zhao, H. Ren, Q. Liang, D. Yuan, S. Xi, C. Wu, W. Manalastas, J. Ma, W. Fang, Y. Zheng, C.-F. Du, M. Srinivasan, Q. Yan, *Nano Energy* **2019**, 62, 94.
[4] L. D. Frederickson, D. M. Hausen, *Analytical Chemistry* **1963**, 35, 818.
